# Environmental Cadmium Exposure Exacerbates Alzheimer’s-like Pathology in a Gut Microbiota-Involved Manner

**DOI:** 10.3390/toxics14080662

**Published:** 2026-07-27

**Authors:** Bao Guo, Junzhuang Chang, Aolu Liu, Minjie Li, Lianghong Guo, Shujun Cheng, Hui Wang, Qian Ba

**Affiliations:** 1 School of Public Health, Shanghai Jiao Tong University School of Medicine, Shanghai 200025, Chinachengsj@126.com (S.C.); 2 Shanghai Chn-Alternative Biotechnology Co., Ltd., Shanghai 201403, China; 3 College of Energy Environment and Safety Engineering, China Jiliang University, Hangzhou 310018, China; 4 School of Environment, Hangzhou Institute for Advanced Study, University of Chinese Academy of Sciences, Hangzhou 310024, China; 5 Science and Technology Innovation Center, Shanghai Municipal Hospital of Traditional Chinese Medicine, Shanghai University of Traditional Chinese Medicine, Shanghai 200071, China

**Keywords:** cadmium, dietary exposure, Alzheimer’s-like pathology, gut microbiota, neuroinflammation

## Abstract

Cadmium (Cd), a ubiquitous environmental toxicant, poses substantial health risks even at low-dose chronic exposures. In this study, we developed a mouse model with chronic low-dose dietary Cd exposure (100 nM CdCl_2_ in drinking water for eight months) to investigate its impacts on cognitive and neuropathological alterations. Behavioral assessments demonstrated that Cd-exposed mice exhibited pronounced deficits in spatial learning, memory retention, and working memory compared with control mice. Histopathological analyses of hippocampus uncovered accelerated Alzheimer’s-like neuropathology, marked by elevated β-amyloid plaque immunoreactivity and tau hyperphosphorylation. Concurrently, neuroinflammatory responses were markedly upregulated, shown as astrocytes activation and pro-inflammatory Th17 cell signatures in parenchyma. Brain transcriptomic profiling revealed extracerebral prostaglandin signaling following Cd exposure, a finding consistent with elevated prostaglandins detected in the gut. Crucially, these outcomes were gut microbiota-involved: antibiotic-mediated microbiota depletion attenuated dietary Cd-enhanced cognitive impairments, neuroinflammation, and prostaglandin upregulation, underscoring the critical role of intestinal microbes in mediating Cd neurotoxicity. Furthermore, in vitro co-culture experiments demonstrated that Cd potentiated prostaglandin production in intestinal epithelial cells—an effect amplified by gut bacterial stimuli. This observation suggests a mechanism under which peripheral prostaglandins may contribute to central inflammatory cascades. Together, these findings support a gut-brain mechanism underlying dietary Cd-exacerbated neurodegeneration and highlight gut homeostasis and prostaglandin signaling as promising therapeutic targets for mitigating Cd-associated neurodegenerative disorders.

## 1. Introduction

Cadmium (Cd) is a well-known environmental contaminant that accumulates in tissues over time, causing chronic harmful consequences. The primary modes of exposure include inhalation, ingestion, and skin contact [1]. Nonsmokers and individuals without occupational Cd exposure are mainly exposed through dietary intake [2,3]. After absorption, Cd accumulates in the kidneys and liver, contributing to persistent toxicity. Cd exposure is linked to a variety of adverse health effects, including kidney impairment, bone demineralization, and gastrointestinal issues [1,4,5,6]. Furthermore, Cd is characterized as a teratogen, mutagen, and carcinogen [7,8,9]. The International Agency for Research on Cancer classifies it as a Group 1 human carcinogen, and the United States Agency for Toxic Substances and Disease Registry lists it among the most toxic substances to human health [10].

The exact mechanisms underlying Cd toxicity remain incompletely clarified, despite its well-established hazardous effects. Recent evidence indicates that chronic exposure to Cd may also be a factor in neurodegenerative illnesses like Alzheimer’s disease (AD) [11,12]. Progressive cognitive decline, memory loss, and language impairment and physical dysfunction are the main symptoms of AD [13,14]. The disease is characterized by amyloid beta (Aβ) plaque aggregation and tau phosphorylation [15]. Such alterations usually develop over a prolonged preclinical period before emergence of overt cognitive dysfunction [16]. Glial activation and pro-inflammatory cytokine secretion are indicators of neuroinflammation, which worsens neuronal injury and accelerates disease progression [17].

Multiple population-based studies have identified Cd exposure as a risk factor for the progression of AD, elevated blood and urinary Cd levels positively correlate with cognitive impairment among older adults [18,19]. Longitudinal studies further reveal that older adults with higher Cd exposure experience more rapid cognitive decline over time [20]. Moreover, urinary Cd levels, a biomarker of long-term Cd burden, have been associated with an increased risk of AD-related mortality [21]. Notably, although AD patients present higher external Cd exposure, the brain Cd levels across distinct brain regions show no significant differences relative to healthy individuals [22]. Consistent with this, more than 95% of ingested dietary Cd is excreted via the gastrointestinal tract, merely 3–5% is absorbed and primarily accumulating in peripheral organs such as the kidneys and liver, with only trace amounts reaches the brain [23]. These suggest that environmental-level dietary Cd exposure hardly promote Alzheimer’s-like pathology through direct neurotoxicity, pointing instead to a potential trans-organ toxic mechanism.

Gut homeostasis is pivotal in mediating the health effects of environmental factors, particularly dietary toxicants. Its disruption critically contributes to Alzheimer’s-like pathogenesis via the gut-brain axis, the bidirectional communication network linking the gastrointestinal tract and the central nervous system [24,25,26,27]. This study aims to investigate the neurotoxic effects of chronic dietary exposure to Cd and the role of gut homeostasis in Alzheimer’s-like pathology. Using mouse models, we examined how Cd exposure affects cognitive function, brain pathology, and neuroinflammation. Through both in vivo and in vitro experiments, we elucidated the contribution of gut microbiota in the neuroinflammation and Alzheimer’s-like progression following Cd exposure.

## 2. Materials and Methods

### 2.1. Animals

Specific pathogen-free (SPF) C57BL/6 male mice (4 weeks old) were purchased from Zhuhai Baishitong Biotechnology Co., Ltd. (Zhuhai, Guangzhou Province, China) and were housed in an SPF environment. The environment was controlled at a temperature of 22–24 °C, humidity of 45–65%, and a 12-h light/dark cycle. All animal procedures were conducted in accordance with ethical guidelines and approved by the Ethics Committee of Shanghai Municipal Hospital of Traditional Chinese Medicine, Shanghai University of Traditional Chinese Medicine (No. 2021122, approval data: 10 November 2021).

### 2.2. Experimental Design for Low-Dose Cadmium Exposure in Mice

Four-week-old male C57BL/6 mice were randomly divided into two groups (*n* = 5): the control group (Ctrl) and the low-dose Cd treatment group (Cd). Sample size was determined according to our previous studies using similar chronic cadmium exposure models [28,29]. The control group received normal drinking water, while the treatment group received drinking water containing cadmium chloride (100 nM). According to standard interspecies dose conversion, this mouse exposure corresponds to an estimated human-equivalent dose of approximately 2.5 μg/kg b.w./week, which closely corresponds to the tolerable weekly intake and the mean intake in humans [28,29,30]. Both groups were fed a standard laboratory chow diet, and mice had free access to food and water. Cadmium exposure was maintained throughout the experiment to establish the low-dose Cd exposure mouse model. Behavioral analyses, immunofluorescence quantification, and Western blot densitometry were performed in a blinded manner whenever possible. The general condition of the mice was observed daily. All experimental procedures were in strict compliance with ethical guidelines for animal research.

### 2.3. Gut Microbiota Depletion Mouse Model

Based on the low-dose Cd exposure mouse model, both the control and Cd exposure groups (*n* = 5) were treated with an antibiotic mixture (Abx: 0.05 g/L ampicillin, 0.025 g/L vancomycin, 0.05 g/L neomycin sulfate, 0.05 g/L metronidazole) in sterile drinking water. The Abx treatment lasted for 7 days, with water being replaced on days 3 or 4 to ensure sufficient intake, followed by an antibiotic-free period lasting for 4 days, alternating this cycle to simulate a gut microbiota-depleted model. Then the mice were treated as described in Section 2.2.

### 2.4. Behavioral Testing

#### 2.4.1. Novel Object Recognition Task (NORT)

NORT was used to evaluate 24-h memory in a 4-day session. The mice were allowed to explore freely for 10 min in an open field apparatus (40 cm by 40 cm by 40 cm polyvinyl chloride arena with white walls and floor) on day 1 (“habituation phase”). The session was videotaped, and the videos were analyzed later for monitoring the exploration and locomotor activity of the mice. On day 2, the mice were given another 5-min habituation phase in the same arena. On day 3, each mouse was placed into the open-field arena and exposed to two identical objects for 5 min (“sample phase”). Exploration counts toward each object were counted, and total exploration counts were used as a control for baseline exploration activity. Twenty-four hours after this sampling phase, the familiar object was substituted with a novel object at the same location, and mice were returned to the arena for 5 min of free exploration (“acquisition phase”) to assess memory. Videos from this acquisition phase were analyzed by at least two experimenters. The recognition index was calculated as a percentage of exploration counts toward each object over two objects, and the recognition index of novel object in comparison with that of the other familiar object was presented. The discrimination index (DI) and recognition index (RI) were calculated using the following formulas: DI (%) = (Time spent with new object − Time spent with old object)/(Total time spent with both objects) × 100%; RI (%) = Time spent with new object/(Total time spent with both objects) × 100%.

#### 2.4.2. Morris Water Maze Test

The Morris water maze test was conducted in a circular tub with a diameter of 120 cm, filled with water to a depth of 40 cm. The maze was surrounded by distinct external visual cues (e.g., laboratory equipment) placed on the walls of the experimental room. These distal cues were kept stable and visible throughout all phases of the experiment. During the training phase, a platform was submerged in the center of one quadrant. Each mouse was given 90 s to find the maze. If unsuccessful, it was gently guided to the platform and allowed to rest for 10 s. The training phase lasted for five consecutive days, with 4 trials per day, and the time to locate the platform was recorded. On the 6th day, the platform was removed, and the time spent in the target quadrant was measured for 3 min. After behavioral testing, the mice’s brains, intestines, and fecal contents were collected.

### 2.5. Western Blotting

Hippocampal and cortical tissues were harvested and homogenized in RIPA buffer (Beyotime) containing the phosphatase and protease inhibitor cocktail (Roche). After homogenizing and centrifuging at 12,000 rpm, 4 °C for 10 min, the supernatant was collected and stored at −80 °C until use. Equal amounts of proteins were resolved by SDS-PAGE gel and then transferred to PVDF membrane by using BIO-RAD PowerPacTM Basic system. After blocking, the membranes were incubated with primary antibody overnight at 4 °C. After washing, the membranes were incubated with anti-rabbit IgG HRP-linked secondary antibody (CST) or anti-mouse IgG HRP-linked secondary antibody (CST) for 70 min at room temperature. The membranes were incubated with enhanced chemiluminescence solution (Tanon). The images were captured by Tanon 5200 multi-imaging system (Tanon Col., Ltd., Shanghai, China). Blot images were quantified by using Gel Pro Analyzer software (v1.1.0). The integrated optical density (IOD) of each band was measured, and the IOD of each band was normalized to the IOD of GAPDH bands in the same lane. Primary antibodies used: APP/β-amyloid (NAB228) mouse mAb (CST), Tau monoclonal antibody (TAU-5) (CST), phospho-Tau (Ser262) polyclonal antibody (CST).

### 2.6. Immunostaining

After blocking with 3% goat serum, frozen sections that were mounted on slides were incubated with primary antibody overnight at 4 °C in a humid chamber. After washing with TBST, sections were incubated with secondary antibody for 2 h at room temperature. After washing with TBST, sections were incubated with DAPI. The images were harvested using confocal microscopy. Primary antibodies used: APP/β-amyloid (NAB228) mouse mAb (CST), Tau monoclonal antibody (TAU-5) (CST), GFAP monoclonal antibody (GA5) (Thermo Fisher).

### 2.7. RNA-Seq

Total RNA was extracted from mouse hippocampus tissues, and its integrity and quantity were assessed via agarose gel electrophoresis and Agilent 5400; all PASS samples were subjected to RNA-seq by Novogene Co., Ltd. (Beijing, China) (*n* = 5 per group). Polyadenylated mRNA was enriched from the total RNA using Oligo(dT) magnetic beads and then fragmented in a buffer containing divalent cations. First-strand cDNA was synthesized using random primers in an M-MuLV reverse transcription system, followed by RNA degradation with RNase H and second-strand synthesis using DNA polymerase I with dNTPs. The resulting double-stranded cDNA was purified, end-repaired, A-tailed, and ligated with sequencing adapters. Fragments of approximately 370–420 bp were selected using AMPure XP beads (Beckman Coulter, Beverly, MA, USA), PCR-amplified, and purified to generate the final library. This library was initially quantified with a Qubit 2.0 Fluorometer (Thermo Fisher Scientific, Carlsbad, CA, USA), diluted to 1.5 ng/μL, and its insert size verified using an Agilent 2100 Bioanalyzer (Agilent Technologies, Santa Clara, CA, USA). Libraries with an effective concentration above 2 nM were pooled according to their concentration and target sequencing depth, then sequenced on an Illumina NovaSeq 6000 (Illumina, Inc., San Diego, CA, USA) to produce 150 bp paired-end reads. Raw sequencing data were filtered to remove adapter sequences, reads containing undetermined bases, and low-quality reads (those with more than 50% of bases having a Qphred score ≤ 20). Clean reads were aligned to the reference genome using HISAT2 (v2.0.5). All samples exhibited exon-mapping rates above 93%. Gene expression was quantified with featureCounts (v1.5.0-p3). Raw read counts were converted to FPKM values to normalize gene expression for sequencing depth and gene length. Finally, differential expression analysis was performed with Limma v3.60.2 (FDR < 0.05, |LFC| > 1.5), and KEGG pathway enrichment along with GSEA was carried out using clusterProfiler (v3.8.1).

### 2.8. ELISA

Mouse intestinal tissue (the jejunum and ileum) was washed with pre-chilled PBS. The tissue was then transferred to a homogenization tube with 2 grinding beads and PBS containing protease inhibitors, and homogenized at 4 °C until no tissue residues remained. The homogenate was centrifuged at 5000 rpm for 10 min at 4 °C, and the supernatant was collected on ice. Protein concentration was quantified using a Bradford assay: 10 μL of each sample or a protein standard (prepared in 1× PBS) was added to a 96-well plate, followed by 300 μL of Bradford reagent per well. Absorbance was measured, and a standard curve was used to calculate the protein concentration; all samples were then normalized to the lowest protein amount. Finally, the levels of PGE2, PGD2, and PGF2α in the tissue were measured using specific ELISA kits.

### 2.9. Non-Targeted Fecal Metabolomics

Fifty milligrams of fecal sample was placed in a sterile 2 mL tube with grinding beads, and 400 μL of extraction solvent (methanol:water, 4:1 *v*/*v*) containing 0.02 mg/mL L-2-chlorophenylalanine was added. The sample was ground for 6 min at −10 °C (50 Hz), ultrasonicated for 30 min at 5 °C (40 kHz), incubated at –20 °C for 30 min, then centrifuged at 4 °C (13,000 *g* for 15 min) to collect the supernatant. Quality control (QC) samples were prepared by pooling equal volumes from all extracts and injected every ten samples to monitor reproducibility. For LC-MS/MS analysis, 2 μL of each sample was separated on an HSS T3 column (100 mm × 2.1 mm, 1.8 μm) (Waters Corporation, Milford, MA, USA) using a gradient with mobile phase A (95% water, 5% acetonitrile, 0.1% formic acid) and mobile phase B (47.5% acetonitrile, 47.5% isopropanol, 5% water, 0.1% formic acid) at 0.40 mL/min and 40 °C. The mass spectrometer, operating in both positive (3500 V) and negative (2800 V) ion modes over an *m*/*z* range of 70–1050, used a source temperature of 400 °C, with MS1 and MS2 resolutions of 70,000 and 17,500, respectively. Raw data were processed using Progenesis QI v4.1 for baseline filtering, peak detection, integration, retention time correction, and alignment, and metabolites were identified by matching MS/MS spectra with public databases before further analysis.

### 2.10. In Vitro E. coli and NCM460 Cells Co-Culture and LC-MS/MS

One microliter of *E. coli* OP50 bacterial solution was taken and put in LB solid medium, and it was incubated at 37 °C overnight. The monoclonal colony was picked with the tips and put into a centrifuge tube with 15 mL of LB liquid medium and incubated at a constant temperature of 37 °C with shaking at 250 rpm. The OD600 was measured during the incubation until the OD600 was about 0.5, and then place into the centrifuge tube for use. DMEM medium containing 100 nM CdCl_2_ and DMEM medium containing *E. coli* was prepared and added to the cells. Then incubated at 37 °C with 5% CO_2_ for 24 h. At the end of the incubation, the cells were washed 2–3 times with 1× PBS solution, then harvested into a 15 mL centrifuge tube and centrifuged at 500 rpm for 5 min. The cells were stored at −80 °C and the levels of prostaglandins (PGB2, PGD2, PGE2, PGF2α and PGJ2) were detected by liquid chromatography tandem mass spectrometry (LC-MS/MS).

In brief, cell samples (*n* = 7) were processed by combined SPE and liquid–liquid extraction. Oasis-HLB SPE cartridges were preconditioned with 1 mL methanol and 1 mL Milli-Q water. Cell homogenate spiked with 5 ng of each internal standard (IS) was loaded, rinsed with 1 mL 5% methanol, vacuum-dried for 20 min, and analytes were eluted with 1 mL methanol, then dried. For liquid–liquid extraction, cell pellets were homogenized in 500 μL methanol (2% formic acid, 0.01 mol/L BHT, 5 ng IS each), vortexed for 5 min, and centrifuged at 12,000 *g*, 4 °C for 10 min. The supernatant was mixed with 700 μL water and 1 mL ethyl acetate, shaken 2 min, and re-centrifuged. Organic layers were pooled after re-extraction of the aqueous phase, evaporated to dryness, reconstituted in 100 μL 30% acetonitrile, and filtered through 0.22 μm nylon centrifugal filters. Separation was achieved on a UPLC BEH C18 column (1.7 μm, 100 mm × 2.1 mm i.d.) at 25 °C with 10 μL injection volume, mobile phase A (water) and B (acetonitrile) at 0.6 mL/min. A 9 min gradient (30–40% B, 0–1.5 min; to 60% B, 1.5–6.5 min; to 80% B held 1 min, 6.5–7.6 min; back to 30% B held 0.2 min, 8.6–8.8 min) separated 32 ARA and 37 ω-3 PUFA metabolites. Targeted detection was performed on a 5500 QTRAP mass spectrometer (AB Sciex LLC, Framingham, MA, USA) with turbo ion spray ESI source, controlled by Analyst 1.5.1. Analytes were monitored in negative MRM mode with 25 ms dwell time; ion source parameters: CUR 40 psi, GS1 30 psi, GS2 30 psi, IS −4500 V, CAD medium, source temperature 500 °C.

### 2.11. Statistic

Data were presented as Mean ± SEM. Mann-Whitney U test (in vivo) and Student’s *t* test (in vitro) were used for statistical analysis. *p* < 0.05 was considered significantly different.

## 3. Results

### 3.1. Chronic Low-Dose Cd Exposure Exacerbated Alzheimer’s-like Phenotypes in Mice

**Figure 1:**
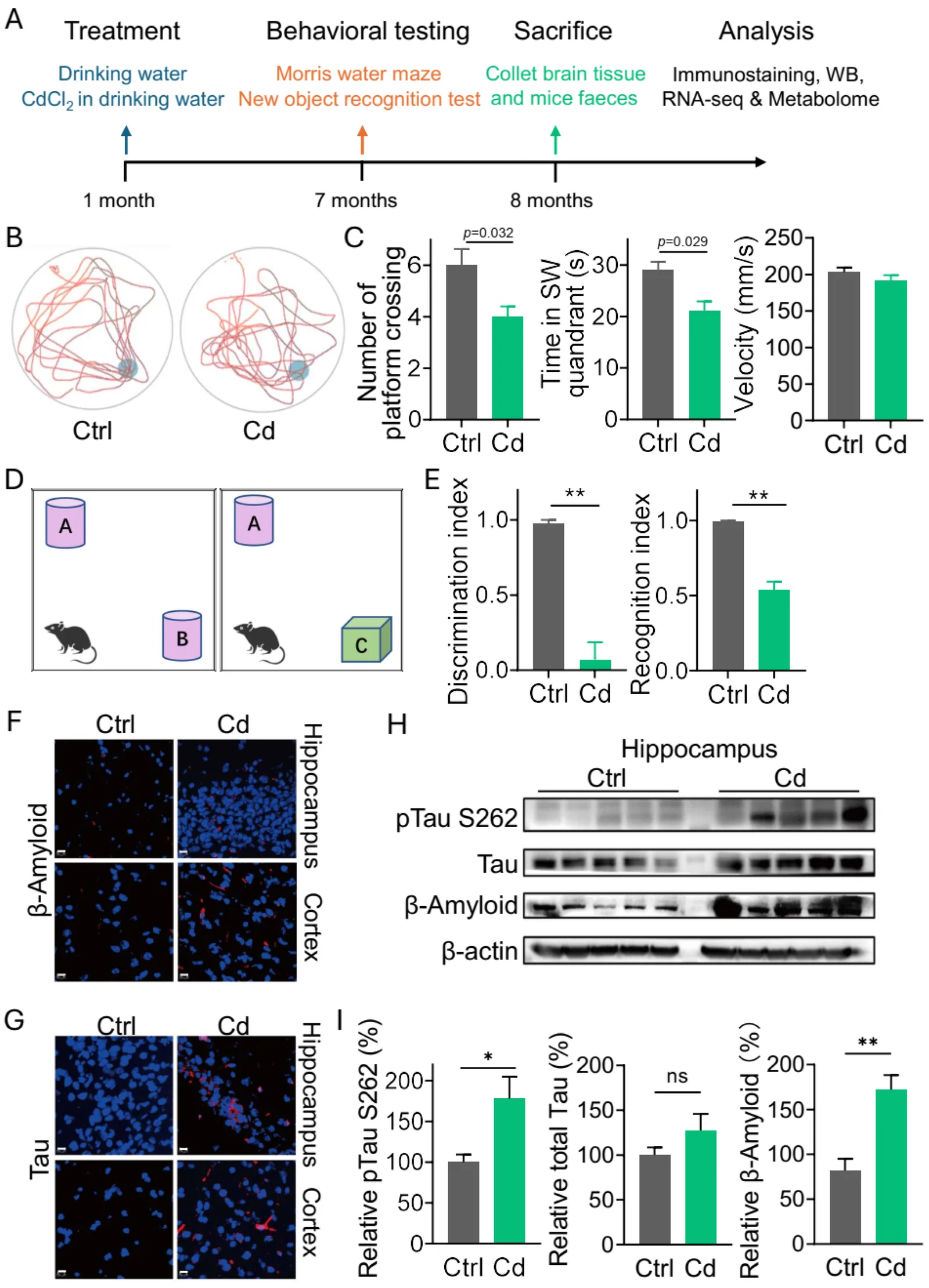
Chronic dietary Cd exposure aggravated Alzheimer’s-like pathology in mice. (**A**). Schematic diagram of long-term and low-dose Cd dietary exposure. (**B**). Representative track images of Ctrl and Cd-treated mice in the probe trial. (**C**). Number of platform crossings of mice in Ctrl and Cd groups during the probe trial; Time spent in target quadrant(s) of mice in Ctrl and Cd groups during the probe trial; Mean swimming speed of mice in Ctrl and Cd groups during the probe trial. (**D**). Experimental diagram for new object recognition test (Objects A and B are old objects, object C is a new object). (**E**). Discrimination index and recognition index of mice in Ctrl and Cd groups during NORT. (**F**). Representative immunofluorescence staining images of β-amyloid (Red) in hippocampus and cortex of mice in Ctrl and Cd groups. (**G**). Representative immunofluorescence staining images of Tau (Red) in hippocampus and cortex of mice in Ctrl and Cd groups. (**H**). Western blotting images of pTau S262, Tau, β-Amyloid and β-actin in the mouse hippocampus of Ctrl and Cd groups. (**I**). Relative quantification of pTau S262, Tau, and β-Amyloid shown in (**H**). Data were shown as Mean ± SEM. * *p* < 0.05; ** *p* < 0.01. Scale bar, 10 μm.

To investigate the health impacts of chronic low-dose dietary Cd exposure, we established a mouse model simulating environmental Cd exposure (CdCl_2_, 100 nM) for 8 months [28,29], while the control group received normal sterile water. Behavioral tests and tissue sampling were conducted, respectively (Figure 1A). To evaluate spatial memory function, Morris water maze (MWM) was performed [31]. In the MWM probe trial, Cd-exposed mice showed reduced platform crossings and shorter dwell time in the target quadrant compared to controls (Figure 1B,C). Swimming velocity remained unchanged (Figure 1C), excluding the possibility of motor deficits. To evaluate working memory and cognitive function, Novel Object Recognition Task (NORT) [32] was performed (Figure 1D). Cd-exposed mice exhibited lower discrimination and recognition indices, indicating impaired working memory (Figure 1E). In addition to phenotypic defects, Cd exposure induced pathological abnormalities. β-amyloid (Aβ) plaque formation and tau protein phosphorylation are the main hallmarks of AD. We found that Cd exposure enhanced β-amyloid and tau pathology. Cd-exposed mice exhibited an increase in Aβ and Tau immunoreactivity both in hippocampus and cortex (Figure 1F,G and Figure S1). Besides, we further confirmed the increase in Tau phosphorylation and Aβ immunoreactivity by Cd exposure through western blotting methods (Figure 1H,I). Thus, our results reveal that chronic low-dose Cd exposure can promote Alzheimer’s-like progression in mice.

### 3.2. Cd Exposure Induced Neuroinflammation in Brain

**Figure 2:**
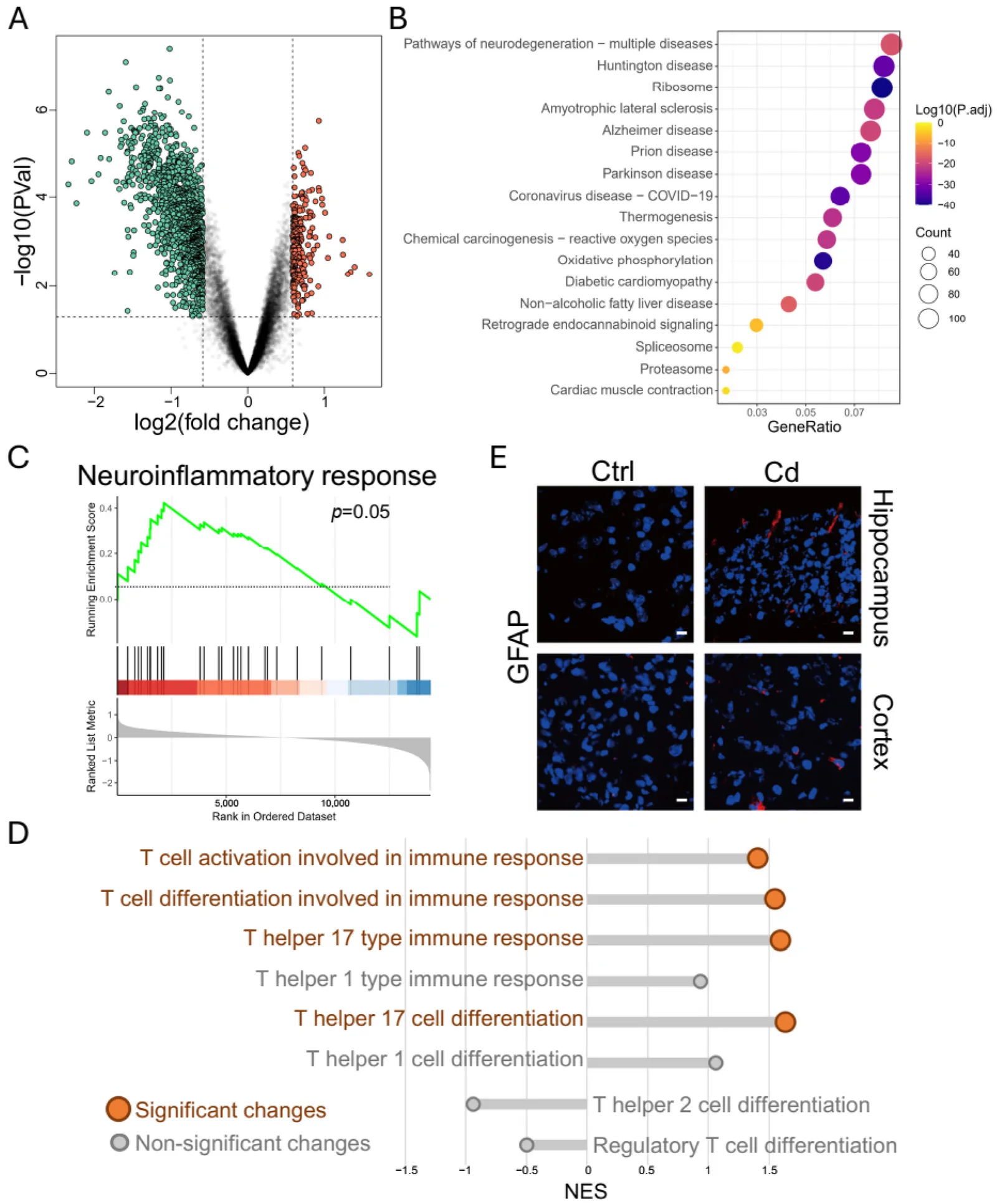
Changes in gene expression in hippocampus of mice after long-term dietary Cd exposure. (**A**). Volcano plot for the differentially expressed genes after Cd exposure. Green dots represent significantly upregulated genes, red dots represent significantly downregulated genes, and grey dots indicate genes with non-significant expression changes. (**B**). KEGG pathway enrichment of the differentially expressed genes. (**C**). Gene Set Enrichment Analysis of the “Neuroinflammatory response”. The green curve denotes the running enrichment score. The color gradient bar at the bottom illustrates the ranked gene list: red corresponds to genes with higher expression in the comparison group, and blue corresponds to genes with lower expression. (**D**). Gene Set Enrichment Analysis of various T cell-related terms. (**E**). Representative immunofluorescence staining images of GFAP (Red) in hippocampus and cortex of mice in Ctrl and Cd groups. Scale bar, 10 μm.

To explore the mechanism of the effects of chronic low-dose Cd exposure on Alzheimer’s-like pathological changes, we collected hippocampus tissues of mice in different treatment groups and performed RNA-seq (Table S1). Principal component analysis (PCA) showed clear separation of gene expression profiles between Cd and Ctrl groups (Figure S2). A total of 1234 differentially expressed genes (DEGs) were identified in Cd-exposed mice (Figure 2A). Strikingly, KEGG enrichment implicated multiple neurodegenerative pathways (such as Huntington, Alzheimer’s, Parkinson’s, ALS, Prion disease) (Figure 2B). We further conducted Gene Set Enrichment Analysis (GSEA) and found that Cd exposure upregulated the neuroinflammatory response gene set (Figure 2C). Neuroinflammation in the brain is associated with various immune cells, including different types of T lymphocytes in the meninges. To further explore the cell types through which Cd exposure induces neuroinflammation, we analyzed the gene sets involved in Cd exposure. Consistent with the upregulation of neuroinflammation, T lymphocytes were significantly activated in the brain tissue of Cd-exposed mice, as evidenced by the significant enrichment terms including *T cell activation involved in immune response* and *T cell differentiation involved in immune response* (Figure 2D). In terms of T cell subtypes, *T helper cell 17 (Th17) type immune response* and *T helper 17 cell differentiation* were significantly increased in the Cd-exposed group, while Th1, Th2, and Treg were not changed (Figure 2D), suggesting that Cd exposure induced neuroinflammation mainly through Th17 differentiation. We also confirmed the activation of the neuroimmune system using immunofluorescence. GFAP, the marker of astrocytes, was increased in hippocampus and cortex in Cd-exposed mice (Figure 2E). In contrast, no such change was observed for the microglia marker IBA1 (Figure S3), suggesting that astrocytes are more sensitive than microglia to Cd-enhanced neuroinflammatory effect. These results suggest that Cd exposure may enhance Alzheimer’s-like phenotype through neuroinflammation, characterized by Th17 and astrocyte activation.

### 3.3. Cd Induced Prostaglandin Production in Intestine

**Figure 3:**
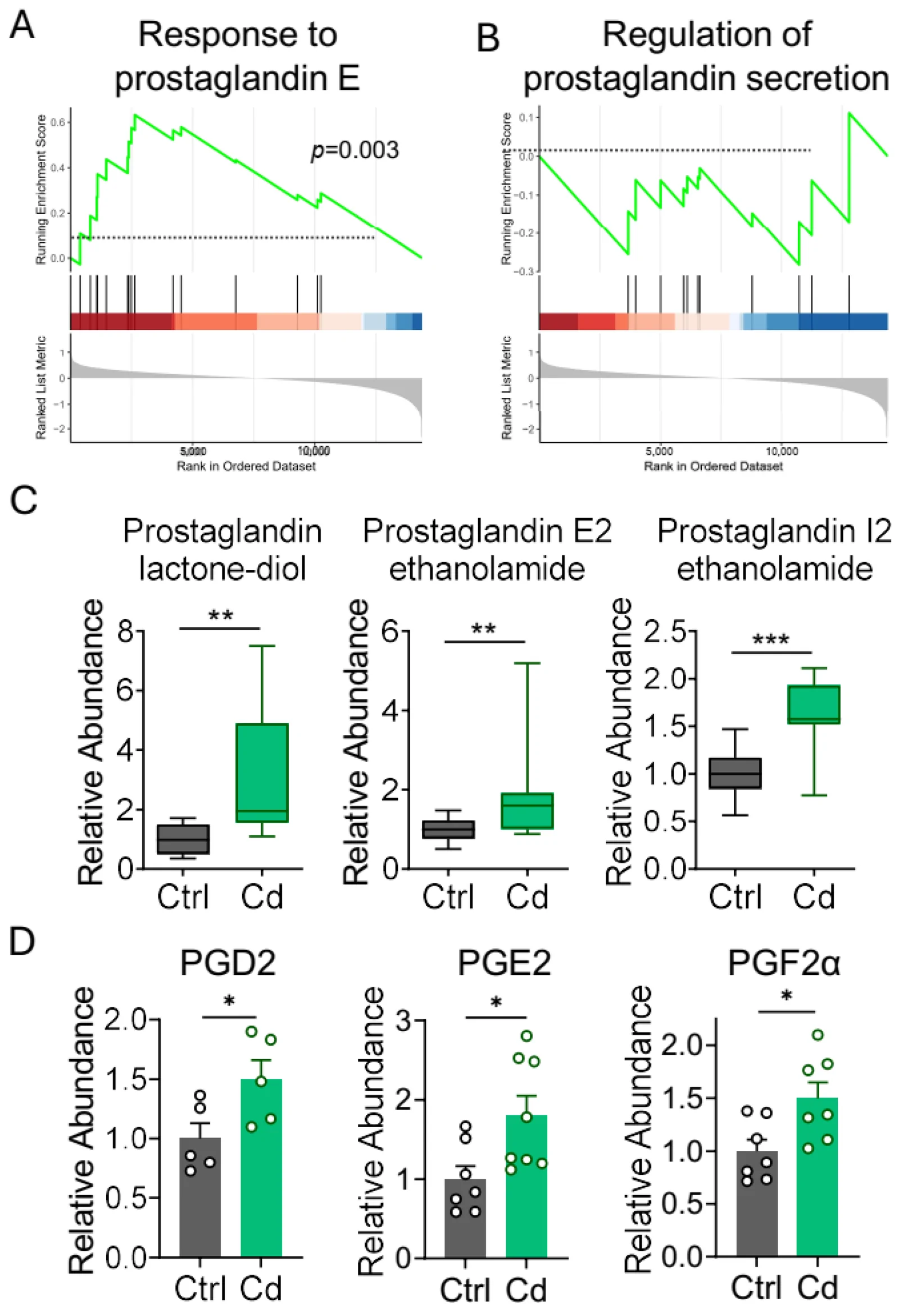
Effects of long-term dietary Cd exposure on intestinal prostaglandin production. (**A**). Gene Set Enrichment Analysis of the “Response to prostaglandin E”. The green curve denotes the running enrichment score. The color gradient bar at the bottom illustrates the ranked gene list: red corresponds to genes with higher expression in the comparison group, and blue corresponds to genes with lower expression. (**B**). Gene Set Enrichment Analysis of the “Regulation of prostaglandin secretion”. The green curve denotes the running enrichment score. The color gradient bar at the bottom illustrates the ranked gene list: red corresponds to genes with higher expression in the comparison group, and blue corresponds to genes with lower expression. (**C**). The relative abundance of prostaglandin derivatives obtained from untarget metabolomics of mouse fecal samples. (**D**). The levels of prostaglandins in intestine of mice. Data were shown as Mean ± SEM. * *p* < 0.05; ** *p* < 0.01; *** *p* < 0.001.

To explore how Cd exposure induced neuroinflammation, we interrogated the transcriptomic data in hippocampus and found that the signaling of *Response to prostaglandin E (PGE)* was significantly elevated in Cd-exposed mice (Figure 3A), which was reported to promote the expansion and differentiation of Th17 subsets in vivo [33]. Interestingly, despite the response being upregulated, *Regulation of prostaglandin secretion* in the brain remained unchanged after Cd exposure (Figure 3B). This suggests that the prostaglandins eliciting brain response likely originated from a trans-organ source rather than being produced locally within the brain microenvironment. Given that dietary Cd can disrupt gut homeostasis to exert toxic effect, we inferred that the elevated prostaglandin response in brain may originate from abnormalities in the intestine. Thus, we performed an untargeted metabolomics test on mouse feces. The results revealed elevated levels of multiple prostaglandin derivatives (prostaglandin lactone-diol, prostaglandin E2 ethanolamide, prostaglandin I2 ethanolamide) in Cd-exposed mice (Figure 3C). Moreover, the levels of PGE2, PGD2, and PGF2α in intestinal tissue were also increased via ELISA assays, further confirming that the prostaglandins were increased in intestine (Figure 3D). These results suggest that chronic low-dose Cd exposure can significantly promote prostaglandin levels in the intestine and induce prostaglandin responses in the brain.

### 3.4. Chronic Dietary Cd Exposure Did Not Enhance Alzheimer’s-like Phenotype in Gut Microbiota Depleted Mice

**Figure 4:**
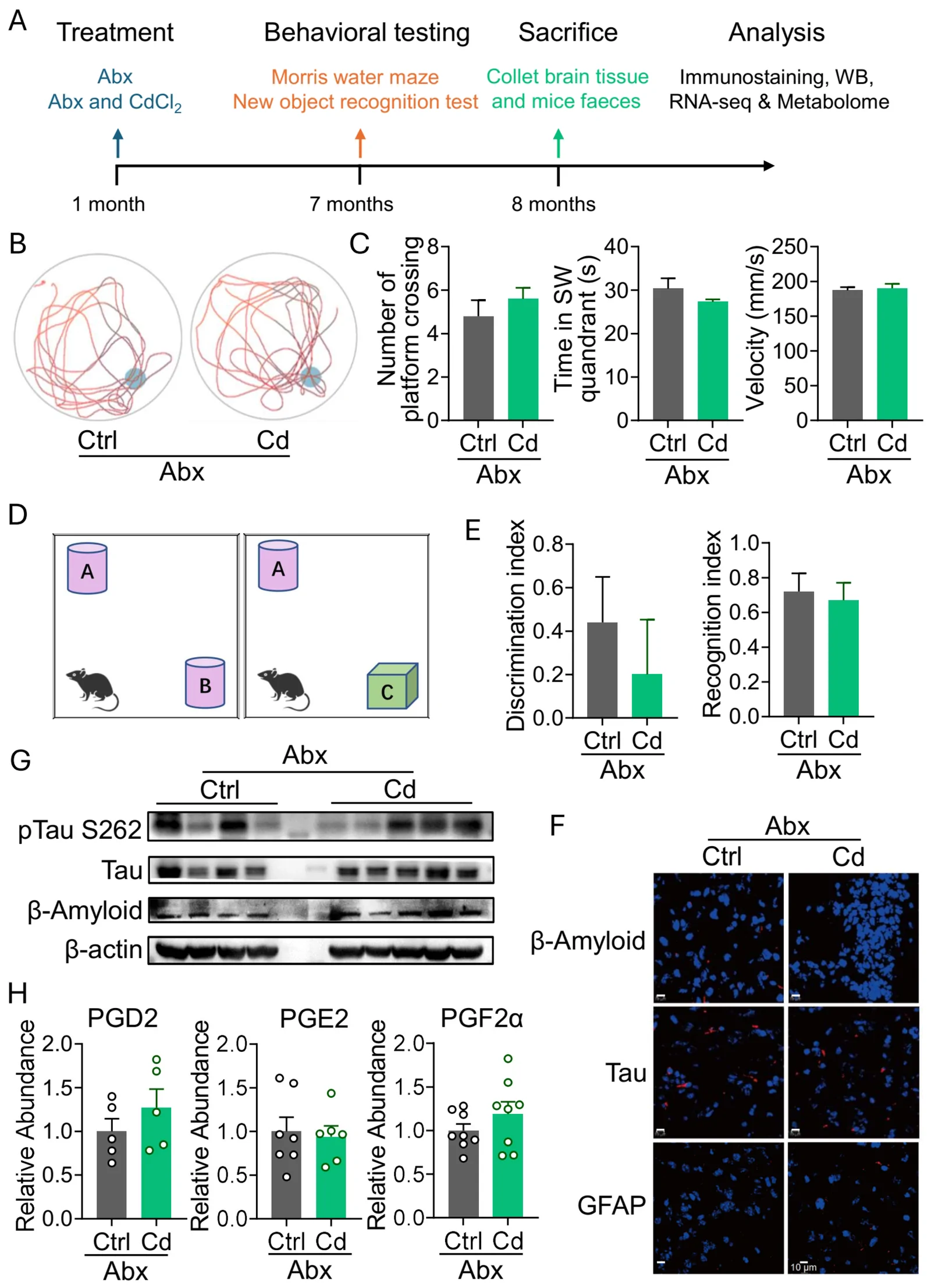
Gut microbiota-depleted mice did not show an exacerbated Alzheimer’s-like phenotype after dietary exposure to Cd. (**A**). Schematic diagram of long-term and low-dose Cd exposure in gut microbiota-depleted mouse model. (**B**). Representative track images of Ctrl and Cd-treated gut microbiota-depleted mice in the probe trial. (**C**). Number of platform crossings of mice in Ctrl and Cd groups during the probe trial; Time spent in target quadrant(s) of gut microbiota-depleted mice in Ctrl and Cd groups during the probe trial; Mean swimming speed of mice in Ctrl and Cd groups during the probe trial. (**D**). Experimental diagram for new object recognition test (Objects A and B are old objects, object C is a new object). (**E**). Discrimination index and recognition index of Ctrl and Cd-treated gut microbiota depleted mice during NORT. (**F**). Representative immunofluorescence staining images of β-amyloid (Red), Tau (Red), and GFAP (Red) in hippocampus of mice in Ctrl and Cd groups. (**G**). Western blotting images of pTau S262, Tau, β-amyloid, and β-actin in hippocampus of Ctrl and Cd-exposed microbiota-depleted mice. (**H**). The levels of prostaglandins in intestine of Ctrl and Cd-exposed microbiota-depleted mice. Data were shown as Mean ± SEM. Scale bar, 10 μm.

To investigate the role of gut microbiota in Cd-aggravated Alzheimer’s-like pathology, we established the microbiota depletion mouse model. Mice were treated with antibiotic cocktails (Abx: ampicillin, vancomycin, neomycin, and metronidazole) in drinking water for 11-day cycles (7 days with Abx followed by 4 days off) throughout the duration of the experiment (Figure 4A). To evaluate spatial memory function, Morris water maze (MWM) was performed. In the MWM probe trial, Cd-exposed gut microbiota-depleted mice did not show reduced platform crossings and shorter dwell time in the target quadrant compared to controls, with no velocity deficiency observed (Figure 4B,C). To evaluate working memory and cognitive function, Novel Object Recognition Task (NORT) was performed (Figure 4D). Cd-exposed gut microbiota-depleted mice exhibited similar discrimination and recognition indices, indicating working memory was not impaired (Figure 4E). These results suggest that gut microbiota clearance can rescue Cd-enhanced Alzheimer’s-like changes. Furthermore, the β-amyloid immunoreactivity and tau disposition did not increase in the hippocampus of Cd-exposed gut microbiota-depleted mice (Figure 4F). Western blotting showed the same phenomenon (Figure 4G). GFAP staining showed no astrocyte activation in hippocampus of Cd-exposed gut microbiota-depleted mice (Figure 4F). ELISA assays for PGE2, PGD2, and PGF2α in mouse intestinal tissue also exhibited unchanged levels (Figure 4H). In summary, these results suggest that gut microbiota play key roles in the neuroinflammation and Alzheimer’s-like changes after Cd exposure.

### 3.5. The Gut Microbes Are Necessary for the Increase of Prostaglandin Production in Intestinal Cells

**Figure 5:**
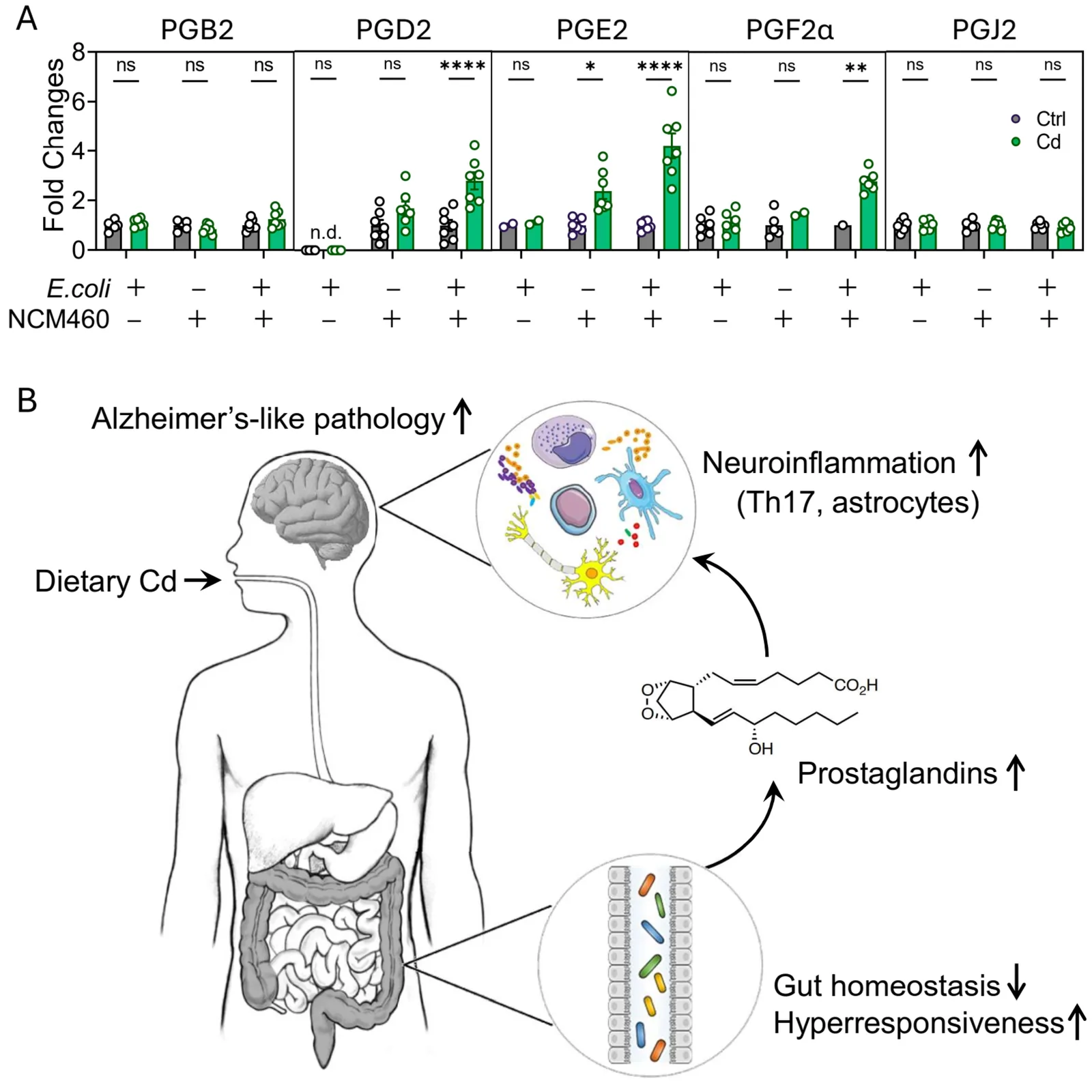
The gut bacteria were necessary for Cd-elevated prostaglandin production in intestinal cells. (**A**). Changes in different prostaglandins in gut bacteria (*E. coli*), intestinal epithelial cells (NCM460), and bacteria-intestinal epithelial cell co-culture model. (**B**). The proposed mechanism diagram of Alzheimer’s-like pathology aggravated by long-term dietary Cd exposure. Data were shown as Mean ± SEM. * *p* < 0.05; ** *p* < 0.01; **** *p* < 0.0001. ↓, downregulation; ↑, upregulation.

To elucidate the role of gut microbes in Cd-induced intestinal prostaglandin production, since Cd exposure primarily occurs in the small intestine, we employed three complementary in vitro experimental models—bacterial monoculture (*E. coli*), intestinal epithelial cells (NCM460), and a bacteria-intestinal epithelial cell co-culture system—to dissect the individual responses of gut bacteria, gut epithelial cells, and their combined interactions to Cd exposure. Following Cd exposure, prostaglandin levels were detected by LC-MS. In the bacterial monoculture, basal prostaglandin levels were negligible, and Cd exposure had no effect (Figure 5A). In NCM460 cells cultured alone, Cd elicited a modest increase in PGE2 production but did not alter levels of other prostaglandins (Figure 5A). In the bacteria-cell co-culture model, co-cultivation with bacteria did not elevate prostaglandin levels in NCM460 without Cd (Figure 5A), consistent with physiological homeostasis. However, upon Cd exposure, the co-culture model exhibited pronounced upregulation of not only PGE2 but also PGD2 and PGF2α (Figure 5A). These results suggest that chronic low-dose Cd exposure renders intestinal epithelial cells hyperresponsive to bacterial stimuli, leading to heightened prostaglandin production that may underlie subsequent neuroinflammatory responses in the brain and contribute to Alzheimer’s-like pathology (Figure 5B).

## 4. Discussion

Chronic exposure to Cd is a major public health concern due to its persistence in the environment and its ability to accumulate in human tissues over time [34,35]. In our study, chronic low-dose Cd ingestion (100 nM CdCl_2_ in drinking water for eight months) aggravated Alzheimer’s-like changes in mice, including deficits in spatial and working memory, increased immunoreactivity of β-amyloid (Aβ), tau protein hyperphosphorylation, and robust neuroinflammatory changes. These observations not only corroborate earlier studies linking Cd exposure to neurotoxicity and cognitive decline [19] but also unveil a novel role of gut microbiota in mediating Cd-induced neurodegeneration.

Our behavioral tests—Morris water maze and novel object recognition—demonstrate that Cd-exposed mice have significant impairments in both spatial and working memory. These cognitive deficits were accompanied by hallmark AD neuropathology, as evidenced by the increased accumulation of Aβ immunoreactivity and tau hyperphosphorylation in the hippocampus and cortex. Similar findings have been reported in previous studies, where Cd exposure was associated with increased oxidative stress and neuronal apoptosis, potentially leading to the formation of neurofibrillary tangles and amyloid deposits [19]. The neurotoxic effects of Cd appear to be mediated partly by its ability to disrupt intracellular calcium homeostasis and impair mitochondrial function, leading to increased reactive oxygen species (ROS) production and subsequent neuronal damage.

Neuroinflammation plays a pivotal role in the progression of AD [36,37]. Our transcriptomic analysis revealed that Cd exposure significantly upregulated genes involved in neuroinflammatory pathways, with a notable increase in markers associated with Th17 cell differentiation. This finding is in line with other reports where Cd exposure resulted in increased expression of inflammatory mediators such as inducible nitric oxide synthase (iNOS) and cyclooxygenase-2 (COX-2) in immune cells. Moreover, immunofluorescence staining showed enhanced expression of glial fibrillary acidic protein (GFAP), indicating astrocyte activation [38], which further supports the existence of a neuroinflammatory environment in Cd-exposed brains.

A unique aspect of our study is the elucidation of the gut microbiota’s role in mediating the neurotoxic effects of Cd. The gut–brain axis is a bidirectional communication system that connects the central nervous system with the gastrointestinal tract. Disruption of gut microbiota homeostasis has been implicated in several neurodegenerative disorders, including AD [39,40]. Our results show that chronic Cd ingestion not only induces brain pathology but also significantly alters the hyperresponsiveness to gut microbes, which may contribute to increased intestinal secretion of prostaglandins. Notably, mice subjected to gut microbiota depletion did not develop the Alzheimer’s-like phenotype upon Cd exposure, suggesting that a functional microbiota is required for the propagation of Cd-induced neuroinflammation. Emerging studies have reported that gut dysbiosis can lead to increased permeability of the intestinal barrier, facilitating the translocation of microbial products and metabolites that can trigger systemic inflammation [41,42]. We have previously reported that chronic dietary Cd exposure can perturb gut microbiota homeostasis (diversity reduction and compositional alteration) and induce compromised intestinal barrier integrity in mice [28,43]. In this study, we further observed increased levels of prostaglandins—especially PGE_2_, PGD_2_, and PGF_2_α in the intestines of Cd-exposed mice. These prostaglandins are potent lipid mediators that have been implicated in inflammatory signaling and can cross the blood–brain barrier to exacerbate neuroinflammation. Our in vitro co-culture experiments further demonstrated that the production of these prostaglandins in intestinal epithelial cells is enhanced by Cd exposure only in the presence of gut bacteria. This implies that Cd might alter bacterial metabolism or stimulate host–microbe interactions that drive prostaglandin synthesis, thereby influencing neuroinflammatory pathways in the brain.

Prostaglandins are derived from arachidonic acid metabolism, and Cd appears to modulate this metabolic pathway. Cadmium has been well documented to disrupt intracellular sulfhydryl homeostasis by depleting free thiols, including glutathione, thereby promoting oxidative stress. Oxidative stress may activate phospholipase A2 and cyclooxygenase pathways, leading to enhanced prostaglandin synthesis [44]. Although thiols and glutathione were not measured in the present study, this mechanism provides a plausible explanation for the increased intestinal prostaglandin levels observed following Cd exposure. Increased prostaglandin levels, in turn, can activate inflammatory cascades via the NF-κB pathway, contributing to the observed neuroinflammation. This mechanism is supported by studies that have shown Cd exposure to enhance COX-2 expression and prostaglandin production in immune cells [45,46]. In our study, the elevated prostaglandin levels in the intestine are particularly significant because they likely serve as peripheral inflammatory signals that affect the central nervous system, thereby linking Cd-induced gut microenvironmental disturbance with Alzheimer’s-like pathology. Several previous investigations have provided insights into the inflammatory and neurotoxic effects of Cd. For example, chronic Cd exposure in mouse macrophages led to an imbalance in redox homeostasis and increased levels of PGE_2_, which contributed to an inflammatory phenotype [45,46]. Cd can modulate eicosanoid production in a dose-dependent manner [47,48]. Cd-induced neurotoxicity in rats highlighted that Cd triggers oxidative stress, inflammation, and apoptosis in the brain, and that these effects could be ameliorated by antioxidants [49,50]. These studies, along with our findings, point to a multifaceted mechanism whereby Cd induces neurodegeneration via oxidative stress, inflammatory mediator release, and disruption of eicosanoid homeostasis.

Gut microbiota play important roles in Cd-exacerbated Alzheimer’s-like changes. It suggests that the gut microbiota act as a critical intermediary between environmental toxicants and brain pathology. Dysbiosis can lead to increased intestinal permeability and the subsequent leakage of pro-inflammatory mediators into circulation. Through systemic circulation, these mediators, such as prostaglandins, can interact with the brain’s immune cells, including microglia and astrocytes, to promote neuroinflammation and synaptic dysfunction. This concept is in line with the growing body of literature emphasizing the importance of the gut–brain axis in neurodegenerative diseases. For instance, several reviews have discussed how alterations in gut microbiota composition may contribute to the progression of AD by modulating systemic inflammation and immune responses [51,52]. Our study adds to this literature by providing experimental evidence that gut microbiota not only modulate but also are necessary for Cd-induced neuroinflammatory responses.

The discovery that depletion of gut microbiota can prevent Cd-induced Alzheimer’s-like pathology suggests new therapeutic strategies. Interventions aimed at restoring or maintaining a healthy gut microbiome—such as the use of probiotics, prebiotics, or dietary modifications—could potentially mitigate the neurotoxic effects of Cd. Moreover, targeting prostaglandin synthesis or blocking specific inflammatory pathways (for example, through COX-2 inhibitors or NF-κB antagonists) might also prove beneficial in preventing or slowing the progression of Cd-exacerbated neurodegeneration. Future studies should focus on identifying the specific bacterial species or communities that are most significantly altered by Cd exposure and that drive prostaglandin production. Metagenomic and metabolomic analyses of the gut microbiota in Cd-exposed models could provide deeper insights into these relationships. Additionally, longitudinal studies in human populations with known Cd exposure could help establish whether similar mechanisms operate in humans and whether gut microbiota modulation can serve as a viable preventive or therapeutic approach for Alzheimer’s and other neurodegenerative conditions. Notably, oxalic acid has been reported to reduce metal bioavailability through the formation of metal–oxalate complexes, thereby representing a potential detoxification mechanism [53]. Future studies are warranted to determine whether these metabolites contribute to Cd-induced gut dysfunction and neurotoxicity.

Previous studies have shown that the toxic effects of Cd may vary with seasonal physiological status in certain organisms, particularly aquatic species undergoing reproductive cycles. Although all animals in the present study were maintained under controlled SPF conditions with constant environmental parameters, seasonal and physiological factors may influence Cd toxicity in other biological systems and should be considered when comparing results across different species and exposure scenarios [54].

This study has several limitations. The proposed transport mechanism from the periphery to the brain requires further validation by measuring serum and cerebrospinal fluid prostaglandin levels. We cannot completely rule out other “gut-brain axis” pathways by which prostaglandins may influence neuroinflammation; for instance, intestinal prostaglandins might stimulate vagus afferent fibers and trigger the release of systemic inflammatory factors such as IL-1β, IL-6, and TNF-α into the circulation, which subsequently activate brain pericytes. Besides, microbiota sequencing, fecal microbiota transplantation, and germ-free mouse studies, additional bacterial species, especially defined microbial consortia, can be included to better capture the complex stimuli of a diverse gut microbiota. The present study employed male C57BL/6 mice to minimize biological variability during long-term exposure; the potential sex-dependent differences in Cd metabolism, immune responses, gut microbiota composition, and Alzheimer’s susceptibility should also be evaluated. Also, internal Cd exposure (e.g., Cd concentrations in blood and tissues) was not determined, as the present work focused on the biological consequences of chronic dietary Cd exposure rather than Cd toxicokinetics. Future studies incorporating these measurements will help better characterize internal dosing and further elucidate the mechanisms underlying Cd toxicity.

## 5. Conclusions

In summary, our study demonstrates that chronic low-dose dietary Cd exposure exacerbates Alzheimer’s-like changes in mice and disrupts gut homeostasis. Although low-dose Cd is not sufficient to trigger inflammation, it renders intestinal epithelial cells hyperresponsive to commensal bacteria and drives excessive prostaglandin production. These peripherally derived prostaglandins may traverse to the brain, amplify neuroinflammatory cascades, and accelerate Alzheimer’s-like pathology. Importantly, depletion of gut microbiota mitigates these effects, underscoring the central role of a dysbiotic gut–brain axis in Cd-exacerbated neurodegeneration. Together, our findings deepen the understanding of environmental risk factors in neurodegenerative diseases and highlight gut homeostasis and prostaglandin signaling as potential intervention targets in Alzheimer’s-like pathology.

## Data Availability

The original contributions presented in this study are included in the article; further inquiries can be directed to the corresponding authors. The sequencing data reported in this study are available in the CNGB Nucleotide Sequence Archive (CNSA: https://db.cngb.org/cnsa; accession number: CNP0009867, accessed on 19 July 2026).

## Supplementary Materials

The following supporting information can be downloaded at: https://www.mdpi.com/article/10.3390/toxics14080662/s1. Figure S1. Representative image of Thioflavin S staining in hippocampus of mice in Ctrl and Cd group. Figure S2. PCA for the gene expression profiling in hippocampus of mice in Ctrl and Cd group. Figure S3. Representative image of IBA1 staining in hippocampus of mice in Ctrl and Cd group. Table S1. RNAseq gene expression matrix.
