# Supplementary material for: Environmental Cadmium Exposure Exacerbates Alzheimer’s-like Pathology in a Gut Microbiota-Involved Manner
Source: Toxics. 2026 Jul 27;14(8):662. doi: 10.3390/toxics14080662 (PMC13517921; doi:10.3390/toxics14080662)
Supplement: Supplementary file 1 [file toxics-14-00662-s001.zip › suppl Figures.pdf]

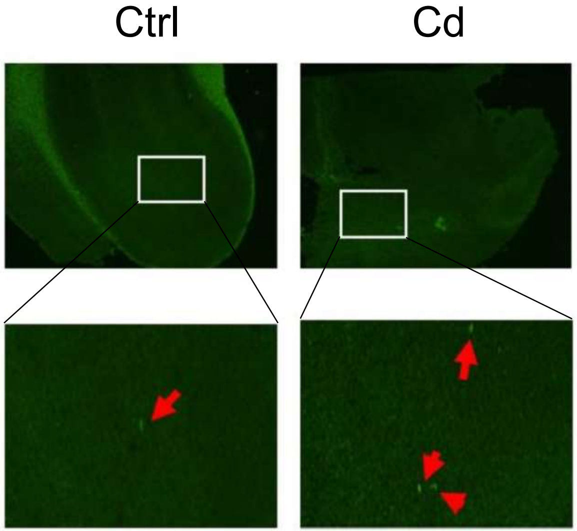

Figure S1. Representative image of Thioflavin S staining in hippocampus of mice in Ctrl and Cd group.

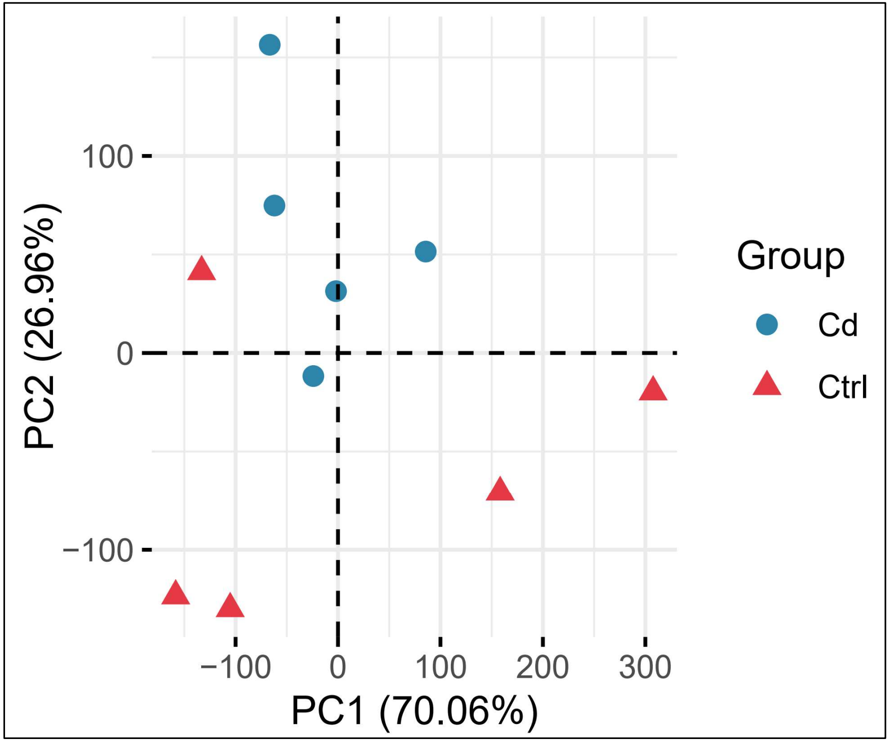

Figure S2. PCA for the gene expression profiling in hippocampus of mice in Ctrl and Cd group.

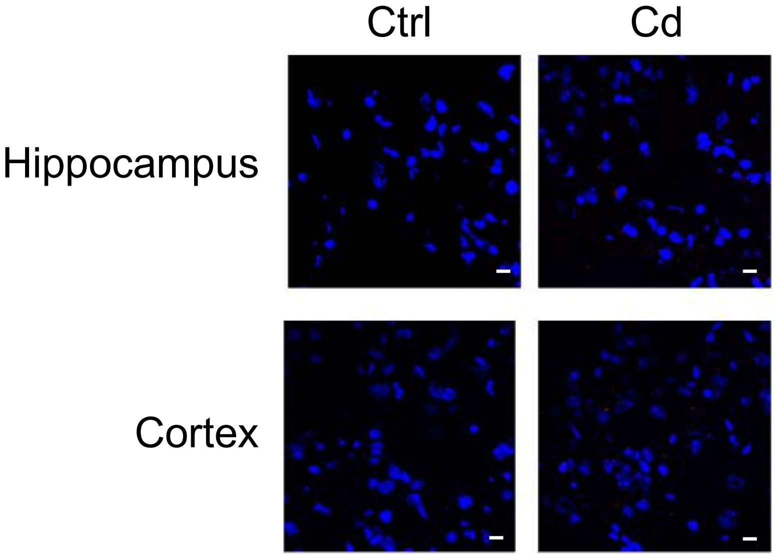

Figure S3. Representative image of IBA1 staining in hippocampus of mice in Ctrl and Cd group.
